# Significant association between polymorphism of the erythropoietin gene promoter and myelodysplastic syndrome

**DOI:** 10.1186/1471-2350-11-163

**Published:** 2010-11-16

**Authors:** Wanlong Ma, Hagop Kantarjian, Ke Zhang, Xi Zhang, Xiuqiang Wang, Clifford Chen, Amber C Donahue, Zhong Zhang, Chen-Hsiung Yeh, Susan O'Brien, Guillermo Garcia-Manero, Neil Caporaso, Ola Landgren, Maher Albitar

**Affiliations:** 1 Department of Hematology/Oncology, Quest Diagnostics Nichols Institute, San Juan Capistrano, CA, USA; 2 Department of Leukemia, M.D. Anderson Cancer Center, University of Texas, Houston, TX, USA; 3 Division of Cancer Epidemiology and Genetics, Center for Cancer Research, National Cancer Institute, National Cancer Institute of Health, Bethesda, MD, USA; 4 Medical Oncology Branch, Center for Cancer Research, National Cancer Institute, National Cancer Institute of Health, Bethesda, MD, USA

## Abstract

**Background:**

Myelodysplastic syndrome (MDS) may be induced by certain mutagenic environmental or chemotherapeutic toxins; however, the role of susceptibility genes remains unclear. The G/G genotype of the single-nucleotide polymorphism (SNP) rs1617640 in the erythropoietin (*EPO*) promoter has been shown to be associated with decreased EPO expression. We examined the association of rs1617640 genotype with MDS.

**Methods:**

We genotyped the EPO rS1617640 SNP in 189 patients with MDS, 257 with acute myeloid leukemia (AML), 106 with acute lymphoblastic leukemia, 97 with chronic lymphocytic leukemia, 353 with chronic myeloid leukemia, and 95 healthy controls.

**Results:**

The G/G genotype was significantly more common in MDS patients (47/187; 25.1%) than in controls (6/95; 6.3%) or in patients with other leukemias (101/813; 12.4%) (all *P *< 0.001). Individuals with the G/G genotype were more likely than those with other genotypes to have MDS (odd ratio = 4.98; 95% CI = 2.04-12.13). Clinical and follow up data were available for 112 MDS patients and 186 AML patients. There was no correlation between EPO promoter genotype and response to therapy or overall survival in MDS or AML. In the MDS group, the GG genotype was significantly associated with shorter complete remission duration, as compared with the TT genotype (*P *= 0.03). Time to neutrophils recovery after therapy was significantly longer in MDS patients with the G/G genotype (*P *= 0.02).

**Conclusions:**

These findings suggest a strong association between the rs1617640 G/G genotype and MDS. Further studies are warranted to investigate the utility of screening for this marker in individuals exposed to environmental toxins or chemotherapy.

## Background

Myelodysplastic syndromes (MDS) are a group of clonal hematopoietic disorders that manifest as ineffective hematopoiesis with hypercellularity in the bone marrow but cytopenia in peripheral blood. MDS can affect each of the three myelopoietic lineages and may progress to acute myeloid leukemia (AML) in some patients[1]. Prior studies suggest that MDS may be induced by certain mutagenic environmental or chemotherapeutic toxins [2,3]; however, the role of genetic factors remains unclear. In fact, the identification of genes that make individuals more susceptible to developing MDS could, for example, provide these high-risk individuals the option of storing frozen bone marrow or hematopoietic stem cells prior to receiving chemotherapy, if they need chemotherapy due to the diagnosis of a cancer in other organ. In addition, identification of genetic factors that enhance susceptibility to MDS, which is a devastating disorder, could suggest mechanistic insights for future interventions.

Genetic variables that potentially associated with MDS risk, include polymorphisms in tumor necrosis factor α and transforming growth factor β, [4,5] as well as variations in the genes related to Bloom syndrome [6]. Single nucleotide polymorphisms (SNPs) have been shown to influence the risk, progression, or pathology of a number of blood or lymph diseases. A recent study demonstrated that the T/T genotype of the rs1617640 SNP, located in the promoter region of the erythropoietin (*EPO*) gene, is significantly associated with diabetic retinopathy and end-stage renal disease in patients with diabetes [7]. The T allele creates a binding site matches the EVI1/MEL1 or AP1 enhancer binding site, leading to increased EPO protein expression. It has been reported that in individuals with the T/T EPO promoter genotype the EPO protein concentration is 7.5-fold higher in vitreous as compared with those with the GG genotype[7]. Individuals with the G/T genotype are expected to be in the middle. Ex-vivo expression experiments showed 25-fold higher expression of EPO in constructs containing the T in the promoter region of the EPO gene[7]. Given that EPO is involved in the control of erythroid and other hematopoietic cell production, [8-11] and because MDS is characterized by impaired production of hematopoietic cells and may respond to EPO therapy, [12-14] we hypothesized that the EPO promoter SNP may show some association with MDS. Here we examined the association of the rs1617640 SNP genotype with MDS in groups of patients with various leukemias and in healthy control subjects.

## Methods

### Patient population

MDS patients (n = 187) were compared to a sample of subjects with AML (n = 257), acute lymphocytic leukemia (ALL; n = 106), chronic lymphocytic leukemia (CLL; n = 97), and chronic myeloid leukemia (CML; n = 353) patients, as well as 95 healthy individuals. Patients with therapy-related MDS were included. The normal control group was volunteers with median age of 33. The ethnic background was not recorded, but females comprised 60% of this group. Because we did not have a complete data on the ethnic background of the MDS patients, we used patients with various types of leukemias as control since they are collected from the same institution and expected to be of the same ethnic background as the MDS patients. Complete clinical data was available for a subset of patients with AML and advanced MDS (n = 182 and 114, respectively). All these patients with clinical data had de novo AML and MDS. All MDS patients with clinical data had advanced disease (platelets <100000/μL, hemoglobin <8, or WBC < 1000/μL). This group of patients was classified according to the French-American-British (FAB) classification. All AML and MDS patients were treated at MD Anderson Cancer Center with standard therapy based on idarubicine + ara-C.

All samples were collected with written informed consent. Samples were collected and work was approved by Institutional Review Committee.

### rs1617640 EPO SNP genotyping

The SNP genotype was determined for each patient and for normal individuals using TaqMan MGB (minor groove binding) probes for allele discrimination (Applied Biosystems, Foster City, CA). Briefly, the rs1617640 EPO SNP was PCR amplified in the presence of MGB probes specific for the G and T SNP alleles. Bound probes were cleaved by the Taq polymerase in the process of PCR amplification, releasing the reporter dyes. Following PCR, plates were read using the 7900HT Fast Real-Time PCR system, and the data were analyzed using Allele Discrimination software (Applied Biosystems).

### Statistical analysis

Patient characteristics were summarized using standard descriptive statistics for continuous variables and tabulations for categorical variables. Relationships between continuous variables were assessed with Spearman rank correlations. Odds ratios and risk ratios with 95% confidence intervals were calculated for each genotype in various comparisons. Kaplan-Meier plots of complete remission duration were performed separately for each diagnostic group.

## Results

### The G/G genotype is more common in MDS

The distribution of rs1617640 genotypes (G/G, G/T, T/T) in MDS patients differed significantly from those of the control and other leukemia groups (Table 1). Except for the ALL group, which showed a mild increase in the G/T (58.5%) genotype as compared to the control group (P = 0.03), there was no significant difference in genotype distribution between any of the acute and chronic leukemia groups, versus control or combined leukemia groups (Table 1). The P-value in Table 1 was calculated based on comparing the three genotypes. The genotype distribution did differ significantly between control subjects and all non-MDS leukemia patients when considered as a group. Patients with MDS had a higher chance of having the G/G than did normal control subjects and patients with other leukemias (Table 2). Patients with myeloid diseases (AML and CML) and those with CLL also had slightly greater chance of having the G/G genotype than did the healthy control group. The ALL group showed odd ratio (OR) for the G/G genotype of 2.26 (95% CI = 0.83-6.13), which is similar to that of AML 2.11 (95% CI = 0.85-5.22) (Table 2). The OR for ALL vs. AML is 1.07 (CI = 0.55-0.99).

**Table 1:** Distribution of rs1617640 EPO SNP genotype in normal control subjects and patients with various hematologic diseases

		EPO SNP Genotype				**P-value (vs. normal controls)**^**a**^	**P-value (vs. all leukemia samples)**^**a**^
Diagnosis		G/G	G/T	T/T	Total		
**Normal**	n	6	41	48	95		
	%	6.32	43.2	50.5			**0.02**
**MDS**	n	47	73	67	187		
	%	25.1	39	35.8		**<.001**	**<0.001**
**ALL**	n	14	62	30	106		
	%	13.2	58.5	28.3		**0.03**	0.61
**AML**	n	32	115	110	257		
	%	12.5	44.8	42.8		0.1	0.21
**CLL**	n	11	34	52	97		
	%	11.3	35.1	53.6		0.22	0.31
**CML**	n	44	173	136	353		
	%	12.5	49	38.5		0.09	0.1

**Total**	n	154	498	443	1095		
	%	14.1	45.5	40.5	100		

^a^Fisher's exact test.

**Table 2:** Odds ratio (OR) and relative risk (RR) and 95% confidence intervals (CI) for the EPO SNP rs1617640 healthy control subjects and patients with various hematologic diseases

	Odds Ratio		Relative Risk	
	OR	95% CI	RR	95% CI
**MDS vs. ALL**				
G/G	2.2	1.15-4.24	1.28	1.07-1.52
G/T	0.45	0.28-0.74	0.75	0.62-0.90
T/T	1.41	0.84-2.37	1.13	0.95-1.34
**MDS vs. AML**				
G/G	2.36	1.44-3.88	1.55	1.24-1.94
G/T	0.79	0.54-1.16	0.87	0.70-1.09
T/T	0.75	0.56-1.10	0.84	0.67-1.06
**MDS vs. CLL**				
G/G	2.62	1.29-5.33	1.31	1.11-1.54
G/T	1.19	0.71-1.98	1.06	0.89-1.25
T/T	0.48	0.29-0.80	0.77	0.64-0.93
**MDS vs. CML**				
G/G	2.36	1.49-3.72	1.66	1.30-2.11
G/T	0.67	0.47-0.96	0.77	0.60-0.97
T/T	0.89	0.62-1.29	0.93	0.73-1.18
**MDS vs. Normal**				
G/G	4.98	2.04-12.13	1.45	1.26-1.67
G/T	0.84	0.51-1.39	0.94	0.79-1.12
T/T	0.55	0.33-0.90	0.81	0.68-0.97
**ALL vs. AML**				
G/G	1.07	0.55-2.10	1.05	0.66-1.68
G/T	1.74	1.10-2.75	1.48	1.07-2.05
T/T	0.53	0.32-0.86	0.63	0.44-0.91
**ALL vs. CLL**				
G/G	1.19	0.51-2.76	1.08	0.74-1.58
G/T	2.61	1.48-4.61	1.57	1.20-2.06
T/T	0.34	0.19-0.61	0.58	0.42-0.80
**ALL vs. CML**				
G/G	1.07	0.56-2.04	1.05	0.64-1.72
G/T	1.47	0.95-2.27	1.34	0.96-1.89
T/T	0.63	0.39-1.01	0.70	0.48-1.02
**ALL vs. Normal**				
G/G	2.26	0.83-6.13	1.38	1.00-1.90
G/T	1.86	1.06-3.25	1.34	1.02-1.76
T/T	0.39	0.22-0.69	0.62	0.46-0.85
**AML vs. CLL**				
G/G	1.11	0.54-2.30	1.03	0.85-1.24
G/T	1.50	0.92-2.44	1.11	0.98-1.26
T/T	0.65	0.41-1.04	0.89	0.78-1.01
**AML vs. CML**				
G/G	1.00	0.61-1.62	1.00	0.75-1.32
G/T	0.84	0.61-1.16	0.91	0.75-1.09
T/T	1.19	0.86-1.66	1.11	0.92-1.33
**AML vs. Normal**				
G/G	2.11	0.85-5.22	1.18	1.01-1.37
G/T	1.07	0.66-1.71	1.02	0.90-1.16
T/T	0.73	0.46-1.17	0.92	0.81-1.05
**CLL vs. CML**				
G/G	0.90	0.44-1.81	0.92	0.52-1.61
G/T	0.56	0.35-0.90	0.63	0.44-0.92
T/T	1.84	1.17-2.90	1.61	1.13-2.29
**CLL vs. Normal**				
G/G	1.90	0.67-5.36	1.32	0.90-1.93
G/T	0.71	0.40-1.27	0.84	0.62-1.14
T/T	1.13	0.64-1.99	1.06	0.80-1.41
**CML vs. Normal**				
G/G	2.11	0.87-5.12	1.13	1.01-1.27
G/T	1.27	0.80-2.00	1.05	0.95-1.16
T/T	0.61	0.39-0.97	0.90	0.81-1.00

In general, in all leukemia patients the ORs of having the G/G genotype were higher than in healthy control subjects but lower than in MDS patients (Table 2). This raises the possibility that individuals with the G/G genotype truly have greater tendency to develop leukemia, or to an abnormally low proportion of control subjects having the G/G genotype. With either consideration, the G/G genotype is particularly associated with MDS.

### Clinical Correlations

Complete clinical data were available for a subset of MDS (n = 114) and AML patients (n = 182). The characteristics of these patients are detailed in Table 3. As shown in Table 3, the patients in the AML group were typical adult AML patients. The patients in the MDS group had advanced disease with significant number (51%) having refractory anemia with excess blasts in transformation (RAEB-T). All patients were treated uniformly with standard chemotherapy and all patients were de novo. As shown in Table 4, the odd ratio of having the G/G genotype in this group of patients were 5.18 for the MDS group and 2.3 for the AML group as compared with the normal control group. The odds were significantly higher in the MDS than in the AML group, but these odds remain high in the AML patients. The new World Health Organization (WHO) classification scheme classifies patients with a blast count between 20% and 30% as having acute leukemia, while the previous FAB classification considers these patients as having MDS with RAEB-T. We therefore evaluated the G/G genotype in RAEB-T and CMML patients separately from the AML patients classified according to the FAB and from the MDS patients classified according to the WHO (Table 5). As shown in Table 5, 21% of the RAEB-T and 27% of the CMML had the G/G genotype, which suggests that these patients are closer to MDS than to AML. Patients with high and intermediate II score on the International Prognostic Scoring System (IPSS) had a borderline difference in EPO genotype (P = 0.06) as compared with AML, while patients with low and intermediate I IPSS score had significantly more GG EPO genotype (P = 0.03). In addition, we investigated if the presence of dysplasia in the patients with AML is associated with higher rate of G/G genotype. There was no significant difference in EPO genotype between cases with multilineage dysplasia versus no dysplasia (P = 0.07).

**Table 3:** Characteristics of the AML and MDS patients with complete clinical data.

Characteristic	AML, n = 186	MDS, n = 112
Median age, years (range)	61 (17-84)	65 (21-85)
Performance Status		
0-1	127	91
2-4	52	20
Missing	7	1
Cytogenetics,		
Favorable	23	0
Unfavorable	57	68
Intermediate	106	44
Missing		
Median white blood cell count (range) × 10^9^/L	8.95 (0.5-300.5)	2.9 (0.6-131.4)
Median Hemoglobin, g/dL (range)	7.8 (2.5-13.1)	7.95 (3.6-13.1)
Median Platelets × 10^9^/L (range)	54 (4-463)	41.5 (2-307)
LDH (U/L)	930 (262-20701)	636 (245-6285)
FAB classification		
M0-2	98	
M3	13	
M4-5	41	
M6/M7	10	
Missing	24	
RA		3
RARS		3
RAEB		38
RAEB-T		58
CMML		11

**Table 4:** Odds ratio (OR) and relative risk (RR) and 95% confidence intervals (CI) for the EPO SNP rs1617640 in AML and MDS patients with complete clinical data.

	Odd Ratio	95%CI	Risk Ratio	95%CI
**MDS vs AML**				
**T/T**	0.82	0.51-1.34	0.88	0.65-1.20
**G/G**	2.25	1.24-4.09	1.58	1.17-2.14
**G/T**	0.72	0.45-1.17	0.82	0.60-1.11
**MDS vs Normal**				
**T/T**	0.54	0.31-0.95	0.75	0.57-0.99
**G/G**	5.18	2.05-13.12	1.72	1.38-2.13
**G/T**	0.82	0.47-1.43	0.91	0.70-1.18
**AML vs Normal**				
**T/T**	0.66	0.40-1.09	0.87	0.73-1.03
**G/G**	2.3	0.91-5.83	1.25	1.03-1.52
**G/T**	1.13	0.69-1.86	1.04	0.88-1.23

**Table 5:** Distribution of rs1617640 EPO SNP genotypes in patients with CMML and RAEB-T as compared to MDS and AML according to WHO classification.

	AML	MDS (WHO)	RAEB-T	CMML
G/T, n (%)	86 (46.24)	10 (23.8)	27 (46.55)	6 (54.55)
T/T, n (%)	74 (39.78)	20 (45.45)	19 (32.76)	2 (18.18)
G/G, n (%)	26 (13.98)	14 (31.82)	12 (20.69)	3 (27.27)

**Figure 1:**
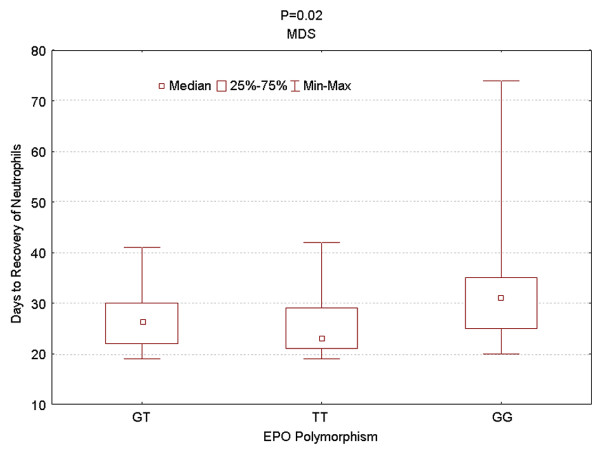
**Longer time to recovery of neutrophils in patients with G/G genotype**.

**Figure 2:**
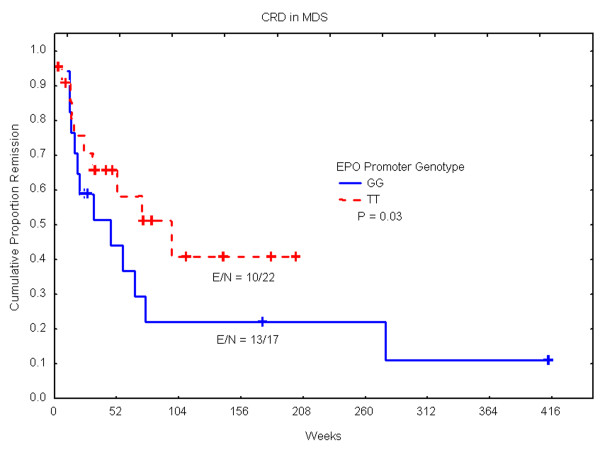
**Shorter complete remission duration (CRD) in patients with G/G genotype as compared with T/T genotype**. Kaplan-Meier plot showing significant difference in CRD between the two genotypes. Abbreviation: N, Number of patients; E, number of events (relapse).

Upon correlating various clinical parameters with rs1617640 genotype, there was no correlation between genotypes and survival, response, age, performance status, cytogenetics, white blood cell count, platelet count, level of hemoglobin, creatinine, beta 2-microglobulin, blood urea nitrogen, and lactate dehydrogenase. However, we found in MDS patients that neutrophils recovery required significantly longer time if patients had the G/G genotype as compared with the other genotypes (*P *= 0.02) (Figure 1). In addition, the MDS group with G/G genotype (n = 22) displayed significantly shorter complete remission duration relative to patients with the T/T genotype (n = 17) (P = 0.03) (Figure 2). However, the number of patients is small since only 39 patients achieved complete response. Despite the small number, we explored the effects of covariates. In multivariate analysis incorporating cytogenetic grouping with EPO polymorphism, EPO polymorphism was no longer significant while cytogenetic grouping was independent predictor of early relapse. Irrespective, this observation suggests that the genotype of the rs1617640 EPO promoter SNP has some influence on maintaining normal hematopoiesis after therapy. It is possible that a different strategy of maintenance therapy should be considered in these patients, especially since the G/G genotype of the EPO has been linked to lower levels of EPO expression relative to the T/T genotype [7]. However, due to the small number of patients and due to the analysis of multiple parameters, a confirmation of clinical correlation in larger studies is needed to rule out overfitting.

## Discussion and conclusions

EPO as a growth factor clearly plays a major role in hematopoiesis. The EPO expression is regulated through complex mechanisms and EPO expression is highly controlled due to its known response to hypoxia. The reported influence of the rs1617640 SNP in the promoter region of the EPO gene on its level of expression, most likely, makes this SNP relevant to hematopoiesis as well. Here we report that the G/G genotype of the rs1617640 SNP is highly associated with MDS, not only as compared with normal control group, but also as compared with patients with other types of acute and chronic leukemias. Unfortunately, the ethnic background of the MDS patients is not available, therefore, we analyzed patients seen in the same institution, but presenting with a different types of leukemias as a control. We are comparing MDS patients to AML, ALL, CML, and AML patients seen in the same institutions. Since none of these diseases has ethnic bias, therefore they can be used as a control. While we do not know, at this point, the mechanism in which this SNP can lead to the development of MDS, the remarkable association between the G/G genotype and MDS suggests a relevance to the development of MDS. The fact that the G/G genotype is associated low levels of EPO hormone suggests that low levels of EPO may play a role in the development of MDS. Not all G/G genotype patients develop MDS, therefore, other factors, especially environmental, may cooperate with the low level of EPO in the development of MDS. Unfortunately pre-MDS EPO levels are not available on these patients. Knowing these levels may provide additional information to better understand the mechanism in which EPO SNP plays a role in MDS. The levels of EPO after developing the MDS should also be studies, however, these levels are influenced by other factors related to the known heterogeneity between MDS patients in hemoglobin and white cell count and may not reflect its role in the development of MDS. It is possible that long-term low EPO level prior to the development of MDS disrupts the normal maturation of hematopoietic cells and eventually leads to neoplasia, especially when this is combined with exposing these hematopoietic cells to environmental or therapeutic toxic agents. In addition, EPO protein is a powerful angiogenic factor and it is possible that prior to the development of MDS, there is low angiogenesis in bone marrow leading to disruption in the normal maturation and differentiation of hematopoitic cells. Of course angiogenesis increases after the development of MDS, but this could be driven by other factors.

While we cannot demonstrate relevance for EPO promoter genotype on outcome in patients with MDS, our data suggests that neutrophils recovery is slower in patients with G/G phenotype. The reason for this is unknown, but it is possible that EPO protein contribute to the recovery of the neutrophils. There was no correlation between hemoglobin recovery and genotype despite that the G/G patients express relatively low level of EPO. The EPO promoter genotype in patients with MDS has never been considered when patients with MDS are treated with EPO protein. The recent studies showed that patients with early MDS may benefit from EPO therapy, especially those with low EPO levels[15-17]. Correlating levels of EPO and response to EPO in MDS patients with EPO promoter genotype may provide important information that may help stratifying patients for such therapy. In addition, EPO protein levels have been implicated in MDS as a cofactor that determine the manifestation of the disease[18]. In that, patients with low EPO protein may present with anemia, while patient with high EPO may have bone marrow dysplasia but adequate hemoglobin and present only when the disease is advanced with the presence of significant neutropenia[18].

Clearly more studies are needed for confirmation of our observations, especially with case control and more detailed racial and environmental data as well as further analysis of other risk factors. The prevalence of the G/G genotype in early stage MDS patients who present with isolated anemia vs. those who present with neutropenia should be explores. In addition, studies are needed to determine the effects of EPO promoter genotype on efficacy of therapy in patients treated with EPO alone or those treated with methylation inhibitors and whether EPO should be added to methylation inhibitors in patients with the G/G genotype. There is also a need to explore the role of this SNP in other leukemias. At this point, we can confirm a strong association between the G/G genotype of the rs1617640 EPO promoter and MDS, but further investigations of the biological effects are needed. Our findings could have significant clinical implications. EPO promoter genotype may influence the efficacy of therapy, especially when EPO is used in early-stage MDS patients. Methylation inhibitors, which are used to treat MDS, may influence the EPO promoter region, and the role of the rs1617640 SNP should be investigated in this context. Our findings also raise questions about the role of the EPO genotype in determining the clinical value of EPO therapy for amelioration of anemia and neutropenia in patients with solid tumors. Given the strong association of the G/G genotype of rs1617640 with MDS, analysis of this SNP may prove to be an important screening tool for high risk patients before they are exposed to toxic agents, therapeutically or environmentally. Clearly, the importance of SNP genotyping in MDS and other diseases will only increase as more links to pathology are uncovered. Our novel finding that the EPO promoter SNP rs1617640 is associated with a 5-fold excess risk of MDS is an important step toward a better understanding of the pathogenesis of this complex disease.

## Abbreviations

MDS: Myelodysplastic syndrome; SNP: single-nucleotide polymorphism; *EPO*: erythropoietin; AML: acute myeloid leukemia; ALL: acute lymphoblastic leukemia; CLL: chronic lymphocytic leukemia; CML: chronic myeloid leukemia; RAEB-T: refractory anemia with excess blasts in transformation; WHO: world health organization; FAB: French America and British; CMML: chronic myelomonocytic leukemia

## Competing interests

The authors declare that they have no competing interests.

## Authors' contributions

WM carried out some of the molecular testing and wrote most of the manuscript. HK, contributed to concept and design and provided data. KZ performed statistical analysis. XW carried out molecular testing. CC carried out molecular testing. ACD contributed to the concept and the writing. ZZ contributed to the concept and design. SOB contributed to concept and design and provided data. GGM contributed to concept and design and provided data. NC contributed to concept and design. OL contributed to concept and design. MA conceived of the study, contributed to design, collected and interpreted data, helped in statistical analysis and finalized writing the paper. All authors read and approved the final manuscript

## Pre-publication history

The pre-publication history for this paper can be accessed here:

http://www.biomedcentral.com/1471-2350/11/163/prepub
